# 4-Chloro-*N*-*m*-tolyl­benzamide

**DOI:** 10.1107/S1600536809018236

**Published:** 2009-05-20

**Authors:** Aamer Saeed, Madiha Irfan, Michael Bolte

**Affiliations:** a Department of Chemistry, Quaid-i-Azam University, Islamabad 45320, Pakistan; b Institut für Anorganische Chemie, J. W. Goethe-Universität Frankfurt, Max-von-Laue-Strasse 7, 60438 Frankfurt/Main, Germany

## Abstract

In the title compound, C_14_H_12_ClNO, the dihedral angle between the two aromatic rings is 11.29 (15)°. The crystal packing is stabilized by N—H⋯O hydrogen bonds linking the mol­ecules into chains running along the *c* axis.

## Related literature

For the biological activity of *N*-substituted benzamides and benzanilide derivatives, see Calderone *et al.* (2006 ▶); Beccalli *et al.* (2005 ▶); Yoo *et al.* (2005 ▶); Vega-Noverola *et al.* (1989 ▶); Olsson *et al.* (2002 ▶); Lindgren *et al.* (2001 ▶); Zhichkin *et al.* (2007 ▶). For related structures see: Saeed *et al.* (2008 ▶); Chopra & Guru Row (2008 ▶); Donnelly *et al.* (2008 ▶)
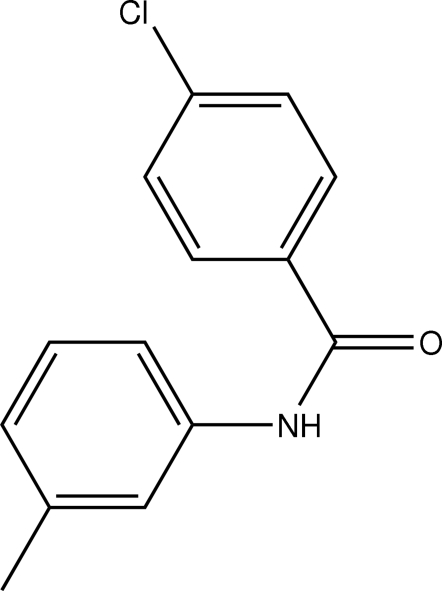


## Experimental

### 

#### Crystal data

C_14_H_12_ClNO
*M* *_r_* = 245.70
Monoclinic, 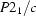

*a* = 13.9721 (14) Å
*b* = 10.1922 (6) Å
*c* = 9.0154 (8) Å
β = 105.415 (7)°
*V* = 1237.67 (18) Å^3^
*Z* = 4
Mo *K*α radiation
μ = 0.29 mm^−1^
*T* = 173 K
0.26 × 0.24 × 0.23 mm

#### Data collection

Stoe IPDSII two-circle diffractometer
Absorption correction: multi-scan (*MULABS*; Spek, 2009 ▶; Blessing, 1995 ▶) *T*_min_ = 0.928, *T*_max_ = 0.936
9469 measured reflections
2193 independent reflections
1787 reflections with *I* > 2σ(*I*)
*R*_int_ = 0.074

#### Refinement

*R*[*F* ^2^ > 2σ(*F* ^2^)] = 0.066
*wR*(*F* ^2^) = 0.178
*S* = 1.04
2193 reflections
160 parameters
H atoms treated by a mixture of independent and constrained refinement
Δρ_max_ = 0.51 e Å^−3^
Δρ_min_ = −0.47 e Å^−3^

### 

Data collection: *X-AREA* (Stoe & Cie, 2001 ▶); cell refinement: *X-AREA*; data reduction: *X-AREA*; program(s) used to solve structure: *SHELXS97* (Sheldrick, 2008 ▶); program(s) used to refine structure: *SHELXL97* (Sheldrick, 2008 ▶); molecular graphics: *PLATON* (Spek, 2009 ▶); software used to prepare material for publication: *SHELXL97*.

## Supplementary Material

Crystal structure: contains datablocks global, I. DOI: 10.1107/S1600536809018236/ya2096sup1.cif

Structure factors: contains datablocks I. DOI: 10.1107/S1600536809018236/ya2096Isup2.hkl

Additional supplementary materials:  crystallographic information; 3D view; checkCIF report

## Figures and Tables

**Table 1:** Hydrogen-bond geometry (Å, °)

*D*—H⋯*A*	*D*—H	H⋯*A*	*D*⋯*A*	*D*—H⋯*A*
N1—H1⋯O1^i^	0.88 (3)	1.99 (3)	2.854 (3)	166 (3)

Symmetry code: (i) 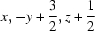.

**Fig. 1.**
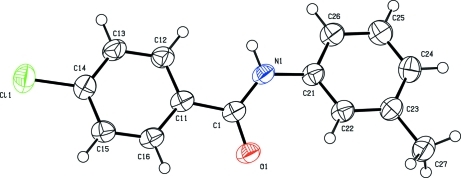
Perspective view of the title compound with the atom numbering scheme; displacement ellipsoids are drawn at the 50% probability level. H atoms are shown as small spheres of arbitrary radii.

## supplementary crystallographic information

### Comment

The benzanilide core is present in compounds with such a wide range of biological activities that it has been called a privileged structure. N-Substituted benzamides are well known anticancer compounds and the mechanism of action for N-substituted benzamide-induced apoptosis has been studied, using declopramide as a lead compound (Olsson *et al.*, 2002). N-Substituted benzamides inhibit the activity of nuclear factor- B and nuclear factor of activated T cells activity while inducing activator protein 1 activity in T lymphocytes (Lindgren *et al.*, 2001). Various N-substituted benzamides exhibit potent antiemetic activity (Vega-Noverola *et al.*, 1989), while heterocyclic analogs of benzanilide derivatives are potassium channel activators (Calderone *et al.*, 2006). *o*-Aryloxylation of N-substituted benzamides induced by the copper(II)/trimethylamine N-oxide system has been studied. *N*-Alkylated 2-nitrobenzamides are intermediates in the synthesis of dibenzo[b,e][1,4]diazepines (Zhichkin *et al.*, 2007) and *N*-Acyl-2-nitrobenzamides are precursors of 2,3-disubstitued 3*H*-quinazoline-4-ones (Beccalli *et al.*, 2005). A one-pot conversion of 2-nitro-n-arylbenzamides to 2,3-dihydro-1*H*-quinazoline-4-ones has also been reported (Yoo *et al.*, 2005). As part of our work on the structure of benzanilides and related compounds, we report here the structure of the title 4-chlorobenzamide derivative, I, Fig 1.

The dihedral angle between the two aromatic rings is 11.29 (15) °. The crystal packing is stabilized by N—H···O hydrogen bonds linking the molecules to chains running along the *c* axis.

### Experimental

4-Chlorobenzoyl chloride (5.4 mmol) in CHCl_3_ was treated with 3-methylaniline (21.6 mmol) under a nitrogen atmosphere at reflux for 3 h. Upon cooling, the reaction mixture was diluted with CHCl_3_ and washed consecutively with aq 1 *M* HCl and saturated aq NaHCO_3_. The organic layer was dried over anhydrous magnesium sulfate and concentrated under reduced pressure. Crystallization of the residue from CHCl_3_ afforded the title compound (81%) as colourless blocks. Anal. calcd. for C_14_H_12_Cl_NO_1: C 68.44, H 4.92, N 5.70%; found: C 68.39, H 4.90, N 5.67%

### Refinement

H atoms were located in a difference map but those bonded to C were geometrically positioned and refined using a riding model with fixed individual displacement parameters [U(H) = 1.2 *U*_eq_(C) or U(H) = 1.5 *U*_eq_(C_methyl_)] and C—H(aromatic) = 0.95Å or *C*—*H*(methyl) = 0.98 Å, respectively. The H atom bonded to N was refined isotropically, N-H 0.88 (3) Å.

### Crystal data

**Table:**

C_14_H_12_ClNO	*F*(000) = 512
*M**_r_* = 245.70	*D*_x_ = 1.319 Mg m^−^^3^
Monoclinic, *P*2_1_/*c*	Mo *K*α radiation, λ = 0.71073 Å
Hall symbol: -P 2ybc	Cell parameters from 10353 reflections
*a* = 13.9721 (14) Å	θ = 2.6–27.8°
*b* = 10.1922 (6) Å	µ = 0.29 mm^−^^1^
*c* = 9.0154 (8) Å	*T* = 173 K
β = 105.415 (7)°	Block, colourless
*V* = 1237.67 (18) Å^3^	0.26 × 0.24 × 0.23 mm
*Z* = 4	

### Data collection

**Table:**

Stoe IPDSII two-circle diffractometer	2193 independent reflections
Radiation source: fine-focus sealed tube	1787 reflections with *I* > 2σ(*I*)
graphite	*R*_int_ = 0.074
ω scans	θ_max_ = 25.0°, θ_min_ = 2.5°
Absorption correction: multi-scan (*MULABS*; Spek, 2009; Blessing, 1995)	*h* = −16→16
*T*_min_ = 0.928, *T*_max_ = 0.936	*k* = −12→12
9469 measured reflections	*l* = −10→10

### Refinement

**Table:**

Refinement on *F*^2^	Secondary atom site location: difference Fourier map
Least-squares matrix: full	Hydrogen site location: inferred from neighbouring sites
*R*[*F*^2^ > 2σ(*F*^2^)] = 0.066	H atoms treated by a mixture of independent and constrained refinement
*wR*(*F*^2^) = 0.178	*w* = 1/[σ^2^(*F*_o_^2^) + (0.1231*P*)^2^] where *P* = (*F*_o_^2^ + 2*F*_c_^2^)/3
*S* = 1.04	(Δ/σ)_max_ < 0.001
2193 reflections	Δρ_max_ = 0.51 e Å^−^^3^
160 parameters	Δρ_min_ = −0.47 e Å^−^^3^
0 restraints	Extinction correction: *SHELXL97* (Sheldrick, 2008), Fc^*^=kFc[1+0.001xFc^2^λ^3^/sin(2θ)]^-1/4^
Primary atom site location: structure-invariant direct methods	Extinction coefficient: 0.018 (5)

### Special details

**Table:**

Geometry. All e.s.d.'s (except the e.s.d. in the dihedral angle between two l.s. planes) are estimated using the full covariance matrix. The cell e.s.d.'s are taken into account individually in the estimation of e.s.d.'s in distances, angles and torsion angles; correlations between e.s.d.'s in cell parameters are only used when they are defined by crystal symmetry. An approximate (isotropic) treatment of cell e.s.d.'s is used for estimating e.s.d.'s involving l.s. planes.
Refinement. Refinement of *F*^2^ against ALL reflections. The weighted *R*-factor *wR* and goodness of fit *S* are based on *F*^2^, conventional *R*-factors *R* are based on *F*, with *F* set to zero for negative *F*^2^. The threshold expression of *F*^2^ > σ(*F*^2^) is used only for calculating *R*-factors(gt) *etc*. and is not relevant to the choice of reflections for refinement. *R*-factors based on *F*^2^ are statistically about twice as large as those based on *F*, and *R*- factors based on ALL data will be even larger.

### Fractional atomic coordinates and isotropic or equivalent isotropic displacement parameters (Å^2^)

**Table:**

	*x*	*y*	*z*	*U*_iso_*/*U*_eq_	
Cl1	0.86033 (5)	0.92615 (6)	0.74007 (9)	0.0588 (3)	
O1	0.41731 (13)	0.85570 (16)	0.23364 (18)	0.0439 (5)	
N1	0.38823 (16)	0.72470 (18)	0.4215 (2)	0.0393 (5)	
H1	0.408 (2)	0.702 (2)	0.519 (3)	0.041 (7)*	
C1	0.44505 (17)	0.80750 (19)	0.3639 (3)	0.0366 (5)	
C11	0.54665 (18)	0.83650 (19)	0.4646 (3)	0.0371 (6)	
C12	0.59229 (19)	0.7638 (2)	0.5951 (3)	0.0435 (6)	
H12	0.5574	0.6930	0.6253	0.052*	
C13	0.68742 (19)	0.7929 (2)	0.6814 (3)	0.0456 (6)	
H13	0.7172	0.7434	0.7711	0.055*	
C14	0.73903 (18)	0.8948 (2)	0.6362 (3)	0.0422 (6)	
C15	0.69544 (19)	0.9691 (2)	0.5060 (3)	0.0413 (6)	
H15	0.7311	1.0387	0.4753	0.050*	
C16	0.6000 (2)	0.94034 (19)	0.4222 (3)	0.0398 (6)	
H16	0.5697	0.9917	0.3343	0.048*	
C21	0.29378 (19)	0.6714 (2)	0.3444 (3)	0.0388 (6)	
C22	0.22613 (19)	0.7341 (2)	0.2241 (3)	0.0404 (6)	
H22	0.2426	0.8166	0.1884	0.049*	
C23	0.13421 (19)	0.6770 (2)	0.1555 (3)	0.0429 (6)	
C24	0.1115 (2)	0.5552 (2)	0.2080 (3)	0.0468 (6)	
H24	0.0499	0.5142	0.1605	0.056*	
C25	0.1784 (2)	0.4941 (2)	0.3291 (3)	0.0488 (6)	
H25	0.1618	0.4118	0.3651	0.059*	
C26	0.2693 (2)	0.5508 (2)	0.3988 (3)	0.0452 (6)	
H26	0.3145	0.5084	0.4826	0.054*	
C27	0.0604 (2)	0.7461 (3)	0.0252 (3)	0.0561 (7)	
H27A	0.0720	0.7202	−0.0732	0.084*	
H27B	0.0686	0.8413	0.0383	0.084*	
H27C	−0.0073	0.7216	0.0264	0.084*	

### Atomic displacement parameters (Å^2^)

**Table:**

	*U*^11^	*U*^22^	*U*^33^	*U*^12^	*U*^13^	*U*^23^
Cl1	0.0465 (5)	0.0523 (5)	0.0671 (6)	−0.0050 (3)	−0.0031 (4)	−0.0004 (3)
O1	0.0528 (11)	0.0441 (9)	0.0317 (9)	−0.0007 (7)	0.0059 (8)	0.0023 (7)
N1	0.0477 (12)	0.0352 (9)	0.0296 (11)	−0.0020 (8)	0.0009 (9)	0.0012 (8)
C1	0.0479 (14)	0.0276 (10)	0.0321 (12)	0.0042 (9)	0.0067 (10)	−0.0024 (8)
C11	0.0481 (14)	0.0277 (10)	0.0340 (12)	0.0040 (9)	0.0081 (11)	−0.0020 (8)
C12	0.0481 (15)	0.0363 (11)	0.0433 (14)	−0.0019 (10)	0.0070 (12)	0.0059 (10)
C13	0.0485 (15)	0.0391 (12)	0.0435 (14)	0.0040 (11)	0.0021 (12)	0.0106 (10)
C14	0.0441 (14)	0.0334 (11)	0.0458 (14)	0.0025 (10)	0.0062 (12)	−0.0051 (10)
C15	0.0522 (14)	0.0306 (11)	0.0409 (13)	−0.0014 (10)	0.0119 (12)	−0.0010 (9)
C16	0.0539 (15)	0.0283 (10)	0.0345 (13)	0.0020 (9)	0.0070 (11)	−0.0005 (9)
C21	0.0468 (13)	0.0322 (11)	0.0343 (12)	−0.0028 (9)	0.0053 (11)	−0.0059 (9)
C22	0.0487 (14)	0.0343 (11)	0.0358 (13)	−0.0011 (9)	0.0069 (11)	−0.0032 (9)
C23	0.0471 (14)	0.0448 (13)	0.0350 (13)	−0.0002 (10)	0.0080 (11)	−0.0066 (10)
C24	0.0490 (15)	0.0410 (12)	0.0478 (15)	−0.0060 (10)	0.0081 (12)	−0.0116 (10)
C25	0.0566 (15)	0.0357 (12)	0.0523 (15)	−0.0047 (11)	0.0111 (13)	−0.0041 (10)
C26	0.0546 (16)	0.0336 (11)	0.0432 (14)	−0.0011 (10)	0.0058 (12)	−0.0013 (9)
C27	0.0510 (17)	0.0587 (15)	0.0500 (16)	−0.0056 (12)	−0.0017 (13)	0.0034 (12)

### Geometric parameters (Å, °)

**Table:**

Cl1—C14	1.734 (3)	C16—H16	0.9500
O1—C1	1.236 (3)	C21—C22	1.390 (3)
N1—C1	1.353 (3)	C21—C26	1.398 (3)
N1—C21	1.426 (3)	C22—C23	1.396 (3)
N1—H1	0.88 (3)	C22—H22	0.9500
C1—C11	1.497 (3)	C23—C24	1.394 (3)
C11—C12	1.393 (3)	C23—C27	1.515 (4)
C11—C16	1.404 (3)	C24—C25	1.383 (4)
C12—C13	1.382 (4)	C24—H24	0.9500
C12—H12	0.9500	C25—C26	1.385 (4)
C13—C14	1.386 (4)	C25—H25	0.9500
C13—H13	0.9500	C26—H26	0.9500
C14—C15	1.393 (3)	C27—H27A	0.9800
C15—C16	1.378 (4)	C27—H27B	0.9800
C15—H15	0.9500	C27—H27C	0.9800
			
C1—N1—C21	127.7 (2)	C22—C21—C26	119.9 (2)
C1—N1—H1	119.2 (18)	C22—C21—N1	123.7 (2)
C21—N1—H1	112.9 (18)	C26—C21—N1	116.4 (2)
O1—C1—N1	123.0 (2)	C21—C22—C23	120.6 (2)
O1—C1—C11	120.3 (2)	C21—C22—H22	119.7
N1—C1—C11	116.7 (2)	C23—C22—H22	119.7
C12—C11—C16	118.3 (2)	C24—C23—C22	119.1 (2)
C12—C11—C1	123.7 (2)	C24—C23—C27	120.5 (2)
C16—C11—C1	118.0 (2)	C22—C23—C27	120.4 (2)
C13—C12—C11	121.1 (2)	C25—C24—C23	120.1 (2)
C13—C12—H12	119.4	C25—C24—H24	119.9
C11—C12—H12	119.4	C23—C24—H24	119.9
C12—C13—C14	119.5 (2)	C24—C25—C26	121.1 (2)
C12—C13—H13	120.2	C24—C25—H25	119.5
C14—C13—H13	120.2	C26—C25—H25	119.5
C13—C14—C15	120.6 (2)	C25—C26—C21	119.2 (2)
C13—C14—Cl1	119.28 (19)	C25—C26—H26	120.4
C15—C14—Cl1	120.04 (19)	C21—C26—H26	120.4
C16—C15—C14	119.3 (2)	C23—C27—H27A	109.5
C16—C15—H15	120.4	C23—C27—H27B	109.5
C14—C15—H15	120.4	H27A—C27—H27B	109.5
C15—C16—C11	121.2 (2)	C23—C27—H27C	109.5
C15—C16—H16	119.4	H27A—C27—H27C	109.5
C11—C16—H16	119.4	H27B—C27—H27C	109.5
			
C21—N1—C1—O1	5.0 (4)	C12—C11—C16—C15	0.8 (3)
C21—N1—C1—C11	−173.24 (19)	C1—C11—C16—C15	−177.3 (2)
O1—C1—C11—C12	−164.2 (2)	C1—N1—C21—C22	−28.5 (4)
N1—C1—C11—C12	14.2 (3)	C1—N1—C21—C26	153.4 (2)
O1—C1—C11—C16	13.8 (3)	C26—C21—C22—C23	−0.9 (4)
N1—C1—C11—C16	−167.84 (19)	N1—C21—C22—C23	−179.0 (2)
C16—C11—C12—C13	0.2 (4)	C21—C22—C23—C24	−0.7 (4)
C1—C11—C12—C13	178.2 (2)	C21—C22—C23—C27	179.5 (2)
C11—C12—C13—C14	−1.0 (4)	C22—C23—C24—C25	1.6 (4)
C12—C13—C14—C15	0.9 (4)	C27—C23—C24—C25	−178.6 (3)
C12—C13—C14—Cl1	−177.17 (19)	C23—C24—C25—C26	−1.0 (4)
C13—C14—C15—C16	0.1 (4)	C24—C25—C26—C21	−0.6 (4)
Cl1—C14—C15—C16	178.14 (18)	C22—C21—C26—C25	1.5 (4)
C14—C15—C16—C11	−1.0 (4)	N1—C21—C26—C25	179.7 (2)

### Hydrogen-bond geometry (Å, °)

**Table:**

*D*—H···*A*	*D*—H	H···*A*	*D*···*A*	*D*—H···*A*
N1—H1···O1^i^	0.88 (3)	1.99 (3)	2.854 (3)	166 (3)

Symmetry codes: (i) *x*, −*y*+3/2, *z*+1/2.

## References

[bb1] Beccalli, E. M., Broggini, G., Paladinoa, G. & Zonia, C. (2005). *Tetrahedron*, **61**, 61–68.

[bb2] Blessing, R. H. (1995). *Acta Cryst.* A**51**, 33–38. 10.1107/s0108767394005726 7702794

[bb3] Calderone, V., Fiamingo, F. L., Giorgi, I., Leonardi, M., Livi, O., Martelli, A. & Martinotti, E. (2006). *Eur. J. Med. Chem.* **41**, 761–767. 10.1016/j.ejmech.2006.03.009 16626840

[bb4] Chopra, D. & Guru Row, T. N. (2008). *CrystEngComm*, **10**, 54–67.

[bb5] Donnelly, K., Gallagher, J. F. & Lough, A. J. (2008). *Acta Cryst.* C**64**, o335–o340. 10.1107/S0108270108012067 18535343

[bb6] Lindgren, H., Pero, R. W., Ivars, F. & Leanderson, T. (2001). *Mol. Immunol.* **38**, 267–277. 10.1016/s0161-5890(01)00060-8 11566320

[bb7] Olsson, A. R., Lindgren, H., Pero, R. W. & Leanderson, T. (2002). *Br. J. Cancer*, **86**, 971–978. 10.1038/sj.bjc.6600136 PMC2364155 11953831

[bb8] Saeed, A., Khera, R. A., Abbas, N., Simpson, J. & Stanley, R. G. (2008). *Acta Cryst.* E**64**, o2322–o2323. 10.1107/S1600536808036313 PMC2960128 21581298

[bb9] Sheldrick, G. M. (2008). *Acta Cryst.* A**64**, 112–122. 10.1107/S0108767307043930 18156677

[bb10] Spek, A. L. (2009). *Acta Cryst.* D**65**, 148–155. 10.1107/S090744490804362X PMC2631630 19171970

[bb11] Stoe & Cie (2001). *X-AREA* Stoe & Cie, Darmstadt, Germany.

[bb12] Vega-Noverola, A. P., Soto, J. M., Noguera, F. P., Mauri, J. M. & Spickett, G. W. R. (1989). US Patent No. 4 877 780.

[bb13] Yoo, C. L., Fettinger, J. C. & Kurth, M. J. (2005). *J. Org. Chem.* **70**, 6941–6943. 10.1021/jo050450f 16095321

[bb14] Zhichkin, P., Kesicki, E., Treiberg, J., Bourdon, L., Ronsheim, M., Ooi, H. C., White, S., Judkins, A. & Fairfax, D. (2007). *Org. Lett.* **9**, 1415–1418. 10.1021/ol070276c 17348669

